# Projective Technique Testing Approach to the Understanding of Psychological Pain in Suicidal and Non-Suicidal Psychiatric Inpatients

**DOI:** 10.3390/ijerph17010284

**Published:** 2019-12-31

**Authors:** Isabella Berardelli, Salvatore Sarubbi, Alessandra Spagnoli, Chiara Fina, Elena Rogante, Denise Erbuto, Marco Innamorati, David Lester, Maurizio Pompili

**Affiliations:** 1 Department of Neurosciences, Mental Health and Sensory Organs, Suicide Prevention Center, Sant’Andrea Hospital, Sapienza University of Rome, 00189 Rome, Italy; isabella.berardelli@uniroma1.it (I.B.); denise.erbuto@gmail.com (D.E.); 2 Department of Psychology, Sapienza University of Rome, 00185 Rome, Italy; sal.sarubbi@gmail.com (S.S.); elena.rogante@gmail.com (E.R.); 3 Department of Public Health and Infectious Diseases, Sapienza University of Rome, 00185 Rome, Italy; alessandra.spagnoli@uniroma1.it; 4 Department of Dynamic and Clinical Psychology, Sapienza University of Rome, 00185 Rome, Italy; chia.fina@gmail.com; 5 Department of Human Sciences, European University of Rome, 00163 Rome, Italy; Marco.Innamorati@unier.it; 6 Psychology Program, Stockton University, Galloway, NJ 19943, USA; David.Lester@stockton.edu

**Keywords:** suicide, mental pain, psychache, psychiatric disorders, suicide risk

## Abstract

Psychological pain is a core clinical factor for understanding suicide, independently from depression. The aim of this study is to assess the role of psychological pain on suicide risk and to evaluate the relationship between psychache and different psychiatric disorders. We conducted the present cross-sectional study on 291 inpatients with a diagnosis of major depressive disorder, bipolar disorder, and schizophrenia. We administered Shneidman’s Psychological Pain Assessment Scale (PPAS) for the assessment of mental pain and the Mini International Neuropsychiatric Interview (MINI) for the assessment of suicide risk. There was a significant association between current psychache and worst-ever psychache and suicide risk in inpatients affected by a depressive disorder, bipolar disorder and schizophrenia. Furthermore, we found a significant difference in current psychache between inpatients with major depressive disorder and inpatients with schizophrenia and in worst-ever psychache between inpatients with bipolar disorder and inpatients with schizophrenia, with lower scores in inpatients with schizophrenia. The assessment of psychache appears to be useful for predicting suicidal risk and should be used routinely for identifying and treating suicide risk in clinical practice.

## 1. Introduction

After dedicating most of his research to the understanding of psychological features of suicidal risk and behavior, Shneidman concluded that suicides are a direct result of immense psychological pain which he named psychache [1]. Psychache is considered to be an acute state of intense psychological pain associated with feelings of shame, humiliation, hurt, anguish, despair, loneliness, fear, and dread which contribute to creating a painful, inner experience conceptualized as a perturbed state [1].

When psychological pain overcomes an individual’s threshold of tolerance, it directly leads to suicide because death is seen as the only means of escape from this unbearable pain [2]. Suicide, therefore, is not an active movement toward death but a way to escape from intense suffering. Several features interfere with one another in the mind of suicides including heightened inimicality (acting against the individual’s best interest), exacerbation of perturbation (refers to how disturbed the individual is), increased constriction of intellectual focus, tunneling or narrowing of the mind’s focus (dichotomous thinking), and the idea of cessation (the insight that it is possible to stop consciousness and put an end to suffering) [3]. Psychache can cause constriction, a narrowing of one’s view of things. It can also lead to perturbation, defined as unrest that causes one to feel like doing something to alleviate the uneasiness one feels. Shneidman considered psychache to be “the introspective recognition of perturbation,” referring to one’s inner turmoil or being upset or mentally disturbed, which may be seen as a continuum from tranquil and serene to frenzied, hypermanic, and wildly disturbed [4,5]. The combination of the press, stress, or pressure, and the weight one feels (perturbation and psychache) fit into what is known as the cubic model of suicide. In the “cubic model” defined by Shneidman [6], psychache is defined as one of three essential dimensions when individuals are considering suicide.

Shneidman [4] proposed the Psychological Pain Assessment Scale (PPAS) as a measure of inner pain. In the PPAS, the author reported the following definition of mental pain: “Psychological pain is not the same as physical pain. It is how one feels like a person; how one feels with one’s mind. It refers to how much pain one feels like a human being. It is mental suffering; a kind of mental torment. This state, literally called mental pain, refers to those conditions that make us feel bad psychologically. It is the pain of feeling guilty, of feeling shame, humiliation, loneliness or having lost something important, or the sadness of growing old or dying in poor conditions or similar things. When this pain is perceived, his inner reality is undeniable”. The PPAS asks several questions about the level of current and worst-ever psychache using a linear rating scale and a checklist for the emotions experienced, along with pictorial stimuli, an application of the technique utilized by Henry Murray in his Thematic Apperception Test [7].

Research has highlighted the central role of mental pain in suicide, while psychological autopsy studies have revealed that affective disorders, substance-related disorders, personality disorders, and psychotic disorders account for most of the diagnoses among suicides [8]. Furthermore, previous research has demonstrated the role of mental pain as a vulnerability factor in patients affected by psychiatric disorders [9,10]. In a previous study using the PPAS, Pompili et al. [11] demonstrated that patients judged to be at risk of suicide reported more psychological pain than patients judged not to be at risk of suicide.

Based on the hypothesis that psychache is associated with the risk of suicide, we conducted the present cross-sectional study with the primary purpose of assessing the predictive power of psychache on suicidal risk. The second aim of this study was to evaluate the relationship between mental pain and psychiatric diagnosis, expecting to find stronger mental pain in inpatients with mood disorders than in inpatients with schizophrenia.

## 2. Materials and Methods

This cross-sectional study was conducted at the University Psychiatric Clinic, Sant’Andrea Hospital, Sapienza University of Rome, between February 2016 and September 2017. The study was approved by the Ethics Committee of the Sant’Andrea Hospital. A total of 291 consecutive inpatients were enrolled in the study. All participants agreed to take part in the study and gave their written informed consent before inclusion.

Of the 291 inpatients: 45% were male, 55% were female, and the mean age was 40.83 years old (SD 13.58); 71 inpatients had a major depressive disorder, 119 had bipolar disorder, and 101 had schizophrenia. Inclusion criteria were meeting the DSM-5 criteria for major depressive disorder, bipolar disorder, or schizophrenia [12], age 18–70 years old, the ability to understand and answer the questions, and not under sedation. Exclusion criteria were a severe form of mental disorder resulting from organic factors and the presence of neurological disease.

### Materials

Inpatients were evaluated using the Italian translation of the Mini International Neuropsychiatric Interview (MINI) [13]. The MINI is a short, structured interview developed jointly by psychiatrists and clinicians in the United States and Europe, for DSM-IV and ICD-10 psychiatric disorders [13]. One section of this instrument is dedicated to the assessment of suicidal risk, with questions about past and current suicidality. The five-item suicidality module of the MINI has been used to establish risk levels. Current suicide risk is established according to cut-off points: 1–5 Low, 6–9 Moderate, ≥10 High. A sixth separate question investigates past suicide attempts.

The Psychological Pain Assessment Scale (PPAS) [4] was administered using an Italian translation. The Italian version was back-translated, and discrepancies between the two versions were corrected. It incorporates a written essay component and requires a trained operator to administer the test and interpret the results. Two trained psychiatrists administered the PPAS.

Page 1 requests personal data (age and sex), presents the purpose of the test and defines psychological pain. The participants are asked to rate their current psychological pain on a scale from 1 (least) to 9 (most). Page 2 of the original PPAS presents ten pictures (five of which are in the shortened version of the scale used here), and respondents are requested to rate the psychological pain experienced by the main character in each picture on a scale of 1–9. The five pictures represent different life scenes (First Steps, The Strike, Departure for War, Adam and Eve, and Thoughtful Woman). The sum of these ratings is calculated. Page 3 asks the respondent to rate the worst psychological pain they have ever experienced on a scale of 1–9, and then to check which of 28 feelings were prominent at that time. Page 4 requests respondents to provide an essay describing their time of worst-ever psychological pain, which we discussed with the patient during the interview, but we did not include it in the statistical analysis given the qualitative nature of the material. Respondents were also asked whether they have ever attempted suicide and to rate the lethality of the most lethal attempt from “very low” to “extremely high”.

## 3. Results

Descriptive data (sex, diagnosis, suicidal risk, and prior suicidal behavior) for the sample are shown in Table 1.

Descriptive statistics for the ratings of psychache are presented in Table 1. Suicide risk, assessed with the MINI, was classified into three subgroups based on the intensity of suicidal ideation and behavior (low, moderate, and high risk): 160 inpatients (55%) had low suicide risk, 33 (11.3%) had moderate suicide risk, and 98 (33.7%) had high suicide risk. Of the 291 inpatients, only 109 inpatients reported a previous suicide attempt as investigated with the sixth separate question of the MINI.

Descriptive statistics are presented as percentages, means, medians, and SDs and are shown in Table 2. The data were checked for normality before applying appropriate statistical tests. Non-parametric tests were used, such as the Kruskal-Wallis Rank Sum Test, and the 5% level of statistical significance. For posthoc analysis, we used Dunn’s test, and the *p*-values were adjusted for multiple statistical tests with the Bonferroni method. The results showed that, in our sample, the rating of current psychache was high (M = 6.1 ± 2.8) on a scale of 1–9, but lower than worst-ever psychache (M = 8.5 ± 1.3) (Table 2). It appears that inpatients had experienced severe psychache and were still suffering from it.

Comparing inpatients with Low, Moderate, and High suicide risk, the results demonstrated that there were significant differences in both current psychache and worst-ever psychache in these three groups Post-hoc comparisons showed that the significant difference was between those inpatients with low suicide risk and those with high suicide risk, demonstrating that inpatients with high suicidal risk reported greater current psychache (M = 6.9 ± 2.4; *p* < 0.001) than inpatients with Moderate (M = 6.6 ± 2.5) and Low suicidal risk.(M = 5.5 ± 2.9) The data concerning worst-ever psychache varied less in the three groups, suggesting that current psychache is more implicated in suicidal risk (Table 3).

The three groups did not differ in the ratings of the psychache portrayed in the five pictures (Table 3).

Comparing the three diagnostic groups, composed of inpatients affected by Depression, Bipolar Disorder, and Schizophrenia (see Table 4), significant differences were found for both current psychache and worst-ever psychache. Post-hoc comparisons showed that the schizophrenic inpatients had significantly lower current psychache and worst-ever psychache than the depressed and bipolar inpatients. Ratings of current psychache in Depressed inpatients was high (M = 7.1 ± 2.02; *p* < 0.01), lower than worst-ever psychache (M = 8.7 ± 0.56; *p* < 0.001); the rating of current psychache in Bipolar inpatients was 6.0 ± 3.0 and the rating of worst-ever psychache was 8.7 ± 1.2, while the rating of current psychache in inpatients with schizophrenia was 5.5 ± 2.8 and the rating of worst-ever psychache was 8.2 ± 1.7. No differences were found for the images of the PPAS in the three group.

## 4. Discussion

Shneidman’s theory of suicide [1] views suicide as a response to overwhelming mental pain. In the present study, we demonstrated the relation between psychache and suicide risk, confirming the results of previous studies [11]. Pompili et al. [11] reported high levels of both current psychache and worst-ever psychache in 88 psychiatric patients, indicating that patients at risk for suicide reported more psychological pain than patients without suicidal risk. In the present study, we found that current psychache and worst-ever psychache were both associated with high suicide risk. The results demonstrated that, in the three different groups, no significant differences were found in the results of the images of PPAS, suggesting that, in our sample, all the patients do not differ as regards the evocation of pain on the observation of images. Projective assessment may offer an evaluative approach in providing useful insights into the dynamics of the inner world experience of patients’ mental pain. However, while the use of projective tests for identifying suicidal ideations is well established, further studies seem necessary to evaluate the usefulness of projective tests [14].

These results show that, regardless of the type of psychiatric diagnosis, the assessment of current psychache and worst-ever psychache is useful for predicting suicidal risk as demonstrated in previous studies on clinical and non-clinical samples [15,16,17]. Caceda et al., in a clinical sample of 62 depressed patients versus 20 healthy controls, investigated the relation between psychological pain and suicidal ideation [15]. The authors, despite the fact that assessed mental pain using alternative assessment tools, found that intense psychological pain was associated with a recent suicide attempt (within 72 h). Leenaars and Lester [16], in a study of 127 undergraduate students at a state college, found that current psychache was associated with prior suicidal ideation and that worst-ever psychache with prior suicidal ideation and suicide attempts. Lester [17], in a sample of 51 students, reported that current and worst-ever psychache were associated with depression and with a history of suicidal ideation. Neither of these two studies, however, studied samples of psychiatric patients.

In the present study, the intensity of psychache differed across the three main psychiatric diagnoses. Inpatients diagnosed with schizophrenia had lower current and worst-ever psychache scores than inpatients diagnosed with major depression or bipolar disorder. These results could be explained by the fact that several neurocognitive deficits have been described in patients with schizophrenia and are now considered to be the main features of the disorder [18,19,20,21]. A recent study by Comparelli et al. [22] demonstrated that schizophrenic patients with suicide ideation had several psychopathological and neurocognitive alterations. We suppose that, in inpatients affected by schizophrenia, these alterations could explain, in part, the low scores on the PPAS scale obtained in our study. However, further research is needed to investigate this possible association.

Recently, Pompili [3] suggested that a phenomenological view can be a fundamental tool for psychiatrists in order to explore mental pain as a main ingredient of suicide beyond any diagnostic criteria. Considering suicide risk as a symptom of a given psychiatric disorder impairs the exploration of the human suffering of suicidal individuals, without which suicide prevention appears unlikely. On the contrary, as Shneidman believed, exploring mental pain and comparing such pain between patient and clinician may help in bridging the gap in the communication of negative emotions. New perspectives in modern psychiatry seem in line with such understanding. For instance, DSM-5 states “Diagnosis of a mental disorder should have clinical utility” but “the diagnosis of a mental disorder is not equivalent to a need for treatment. Need for treatment is a complex clinical decision that takes into consideration symptom severity, symptom salience (e.g., the presence of suicidal ideation), the patient’s distress (mental pain)” [23] (p. 20).

The present study had a number of limitations. A sound measure of psychache would probably be a multi-item scale, and future research should explore the use of alternative scales that assess psychache [24]. The present sample sizes were small, and a follow-up study is needed to explore the predictive power of psychache for suicidal ideation and behavior. A control population without psychiatric disorders would have allowed us to understand better the results concerning the evocation of pain in observing the different images of the PPAS.

## 5. Conclusions

The evaluation of psychache is of fundamental clinical importance because it may be the core feature of vulnerability for suicidal ideation, suicidal attempts, and suicide. Several studies have indicated that mental pain will become critical when the person has no ability to regulate the emotional pain experienced [24]. In line with our results, we propose that, if mental pain is assessed and controlled, the effect of other risk factors for suicide would be largely attenuated. Our results document the link between psychache, suicide risk and different psychiatric disorders and suggest the usefulness of assessing mental pain routinely in psychiatric inpatients for identifying and treating suicide risk in clinical practice.

## Figures and Tables

**Table 1:** Demographic data.

		N	%
**Sex**	Male	131	45
	Female	160	55
	Total	291	100
**Diagnosis**	Major depression	71	24.4
	Bipolar disorder	119	40.9
	Schizophrenia	101	34.7
	Total	291	100
**Suicide risk**	Low	160	55
	Moderate	33	11.3
	High	98	33.7
	Total	291	100
**Suicide attempts lifetime**	No	182	62.5
	Yes	109	37.5
	Total	291	100

**Table 2:** Descriptive statistics.

	N	Mean	Sd	Median	p25	p75	Min	Max
Current psychache	291	6.1	2.8	7	4	9	1	9
First Steps img 1	291	3.7	2.9	3	1	6	1	9
The strike img 2	291	5.9	2.5	6	4	8	1	9
Departure for the War img 3	291	7.3	2.2	8	6	9	1	9
Adam and Eve and the corpse of Abel img 4	291	7.0	2.5	8	6	9	1	9
Thoughtful woman img 5	291	4.6	2.8	5	2	7	1	9
Worst-ever psychache	291	8.5	1.3	9	9	9	1	9

**Table 3:** Psychache by Suicide risk Kruskal-Wallis Rank Sum Test.

	Low ^a^		Moderate ^b^		High ^c^		χ^2^	*p*	Post-hoc
	N	Mean (SD)	N	Mean (SD)	N	Mean (SD)			
Current psychache.	160	5.5 (2.9)	33	6.6 (2.5)	98	6.9 (2.4)	17.92	<0.001	A < C
First Steps	160	3.6 (2.6)	33	3.3 (2.7)	98	4.1 (2.9)	3.49	0.17	-
The strike	160	6.0 (2.6)	33	5.7 (2.5)	98	6.0 (2.5)	0.415	0.81	-
Departure for the War	160	7.4 (2.2)	33	6.8 (2.3)	98	7.5 (2.2)	4.15	0.12	-
Adam and Eve	160	6.8 (2.8)	33	6.7 (2.4)	98	7.5 (2.2)	5.51	0.06	-
Thoughtful woman	160	4.4 (2.7)	33	5.0 (2.9)	98	4.8 (2.9)	2.50	0.29	-
Worst-ever psychache	160	8.3 (1.6)	33	8.5 (1.0)	98	8.9 (0.4)	7.01	<0.05	A < C

Current psychache a < c, *p* < 0.001; worst-ever psychache a < c, *p* < 0.05.

**Table 4:** Psychache scale by diagnosis Kruskal-Wallis Rank Sum Test.

	Depression ^a^		Bipolar Disorder ^b^		Schizophrenia ^c^		χ^2^	*p*	Post-hoc
	N	Mean (SD)	N	Mean (SD)	N	Mean (SD)			
Current psychache	71	7.1 (2.02)	119	6.0 (3.0)	101	5.5 (2.8)	13.35	0.001	A > C
First Steps	71	3.4 (2.71)	119	3.6 (2.9)	101	4.0 (3.0)	1.57	0.45	-
The strike	71	5.7 (2.65)	119	6.2 (2.4)	101	5.7 (2.6)	3.24	0.22	-
Departure for the War	71	7.4 (2.09)	119	7.5 (2.1)	101	7.1 (2.3)	3.84	0.15	-
Adam and Eve	71	7.4 (2.25)	119	7.5 (2.2)	101	6.9 (2.1)	5.63	0.06	-
Thoughtful woman	71	4.6 (2.87)	119	4.8 (2.8)	101	4.4 (2.8)	0.852	0.59	-
Worst-ever psychache	71	8.7 (0.56)	119	8.7 (1.2)	101	8.2 (1.7)	16.06	<0.001	B > C

Current psychache a > c, *p* < 0.01; worst-ever psychache b > c, *p* < 0.001.
